# Supplementary material for: Mathematical modeling and computer simulation of needle insertion into soft tissue
Source: PLoS One. 2020 Dec 22;15(12):e0242704. doi: 10.1371/journal.pone.0242704 (PMC7755224; doi:10.1371/journal.pone.0242704)
Supplement: S1 Table — Numbers in red indicate the difference between the predicted and experimentally determined displacement magnitude greater than 0.32 mm (twice the in-plane image resolution). This table lists the numerical values for the results shown in Figs 15 and 16. (DOCX) [file pone.0242704.s001.docx]

**S1 Table.** Predicted (using the MTLED algorithm with the kinematic approach for needle insertion modeling we introduced in this study) and experimentally determined (from the CT images) displacement field magnitude of the beads for the needle insertion to the depth of 5 mm. Numbers in red indicate the difference between the predicted and experimentally determined displacement magnitude greater than 0.32 mm (twice the in-plane image resolution). This table lists the numerical values for the results shown in Fig. 15 and Fig. 16.

| **# of beads** | **Numerical (mm)** | **Experimental (mm)** | **\|Difference\| (mm)** |
| --- | --- | --- | --- |
| 1  2  3  4  5  6  7  8  9  10  11  12  13  14  15  16  17  18 19  20  21  22  23  24  25  26  27  28  29  30  31  32  33  34  35  36  37  38  39  40  41  42  43  44  45  46 | 0.088  0.100  0.117  0.085  0.150  0.153  0.135  0.173  0.056  0.084  0.115  0.122  0.244  0.495  0.141  0.140  0.233  0.499  0.318  0.308  0.190  0.196  0.779  0.287  0.438  0.210  0.686  0.146  0.255  0.110  0.416  0.062  0.231  0.195  0.079  0.165  0.102  0.215  0.119  0.123  0.125  0.151  0.066  0.089  0.081  0.103 | 0.078  0.142  0.143  0.069  0.310  0.263  0.306  0.177  0.440  0.169  0.385  0.407  0.182  0.376  0.279  0.415  0.448  0.274  0.333  0.233  0.485  0.103  0.637  0.376  0.490  0.543  0.754  0.272  0.326  0.324  0.448  0.292  0.421  0.473  0.289  0.547  0.688  0.378  0.202  0.549  0.435  0.572  0.682  0.507  0.145  0.560 | 0.010  0.041  0.025  0.016  0.159  0.109  0.171  0.321  0.383  0.084  0.270  0.284  0.061  0.118  0.137  0.275  0.214  0.225  0.014  0.075  0.295  0.093  0.140  0.088  0.051  0.333  0.069  0.126  0.071  0.213  0.030  0.229  0.189  0.278  0.210  0.382  0.586  0.163  0.082  0.426  0.310  0.420  0.616  0.418  0.064  0.457 |
